# Supplementary material for: A Sustainable Strategy for Solid-Phase Extraction of Antiviral Drug from Environmental Waters by Immobilized Hydrogen Bond Acceptor
Source: Nanomaterials (Basel). 2022 Apr 10;12(8):1287. doi: 10.3390/nano12081287 (PMC9027420; doi:10.3390/nano12081287)
Supplement: Supplementary file 1 [file nanomaterials-12-01287-s001.zip › nanomaterials-1647514-supplementary.pdf]

Supplementary information

A Sustainable Strategy for Solid-phase Extraction of Antiviral Drug from Environmental Waters by Immobilized Hydrogen Bond Acceptor

Hongrui Yang, Chen Wang, Wenjuan Zhu, Xia Zhang, Tiemei Li, Jing Fan*

School of Environment, Key Laboratory of Yellow River and Huai River Water Environment and Pollution Control, Ministry of Education, Henan Key Laboratory for Environmental Pollution Control, Henan Normal University, Xinxiang, Henan 453007, PR China

Table of contents

1. The preparation of deep eutectic solvent of choline chloride and arbidol
2. **The Equation S1.** The ion/molecule fraction distribution formula
3. **Table S1.** The EDS element content of $PS\text{-}CH_2Cl$ (a) and the immobilized-HBA (b)
4. **Table S2**. The melting point of choline chloride, arbidol and choline chloride-arbidol
5. **Table S3.** The ionization degree of arbidol at different pH values
6. **Table S4**. Kinetic parameters for the adsorption of arbidol by the immobilized-HBA at 20°C and pH=5.5
7. **Figure S1.** The Energy dispersive X-ray energy spectrum of $PS\text{-}CH_2Cl$
8. **Figure S2.** The Energy dispersive X-ray energy spectrum of the immobilized-HBA
9. **Figure S3.** FT-IR spectra of $PS\text{-}CH_2Cl$ (a) and the immobilized-HBA (b)
10. **Figure S4.**TGA curves of $PS\text{-}CH_2Cl$ (a) and the immobilized-HBA (b)
11. **Figure S5.** Mid-IR spectra (a) and Far-IR spectra (b) of choline chloride (1), choline chloride-arbidol (2), and arbidol (3)
12. **Figure S6.** Influence of temperature on the extraction efficiency of arbidol. Conditions: $C_{arbidol}$ = 25 mg/L, t = 30 min, pH=5.5
13. **Figure S7.** Adsorption isotherms of arbidol onto the immobilized-HBA fitted by Freundlich model and the Langmuir model / Conditions: t = 30 min, T = 20 °C, $m_{immobilized\text{-}HBA}$=10 mg, $V_{arbidol}$=5ml, pH=5.5.

14. **Figure S8.** The pseudo-first-order model and pseudo-second-order model for the adsorption of arbidol by the immobilized-HBA / Conditions: $C_{arbidol}$ = 300 mg/L, T = 20°C, pH=5.5.
15. **Figure S9.** UV-visible absorption spectra of arbidol (a) and the recovered arbidol (b)
16. **Figure S10.** FT-IR spectra of the fresh (a) and the regenerated (b) immobilized-HBA.
17. **Figure S11.** The structure of arbidol

The preparation of deep eutectic solvent of choline chloride and arbidol

One kind of deep eutectic solvents was prepared by using choline chloride as hydrogen bond acceptor (HBA) and arbidol as hydrogen bond donor (HBD). The DES was prepared by simply mixing choline chloride (HBA) and arbidol (HBD) under stirring at room temperature, and then reacted at 120 °C for 30 min until a uniform and stable liquid was formed. The molar ratio of choline chloride to arbidol was 1:1.

The Equation (S1)

The ion/molecule fraction distribution was calculated by

$$\varphi_{ions} = \frac{1}{[1+10^{(pKa-pH)}]} \qquad \text{(S1)}$$

where φ_{ions} stands for ion fraction, the pKa of arbidol is 6.5.

Table. S1. The EDS element content of PS-CH_2Cl and the immobilized-HBA

Material type	Element	Content (%)
PS-CH_2Cl	C	72.77
	Cl	27.23
immobilized-HBA	C	58.24
	Cl	14.78
	N	8.83
	O	18.15

Table. S2. The melting point of choline chloride, arbidol and choline chloride-arbidol

Chemical	Melting point /°C
choline chloride	305.1
arbidol	134.8
Choline chloride-arbidol(1:1)	88.4

Table S3. The ionization degree of arbidol at different pH values

pH	φ_{ions}	pH	φ_{ions}
3	3.162×10^{-4}	6.5	0.50
4	3.152×10^{-3}	7	0.759
5	3.065×10^{-2}	8	0.969
5.5	9.090×10^{-2}	9	0.997
6.0	0.240		

Table S4. Kinetic parameters for the adsorption of arbidol by the immobilized-HBA at 20 °C and pH=5.5

pseudo-first-order model			pseudo-second-order model		
K_1(min^{-1})	q_e(mg/g)	R^2	K_2(g/(mg·min)	q_e(mg/g)	R^2
0.029	9.77	0.635	8.57×10^{-4}	145.8	0.994

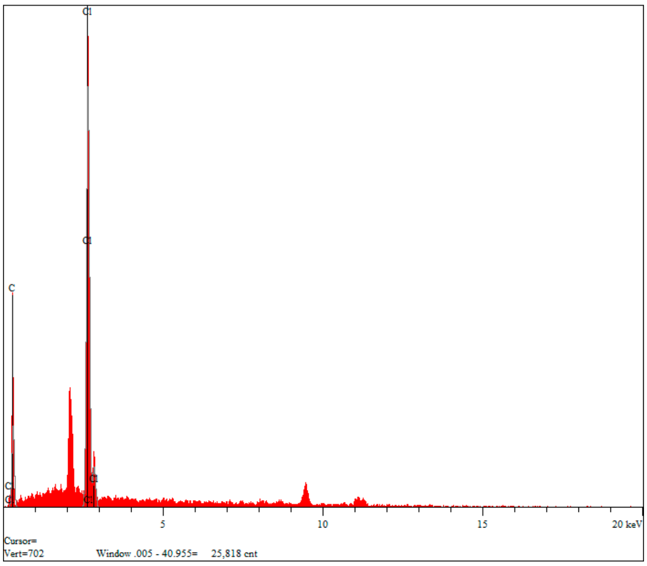

Figure S1. Energy dispersive X-ray energy spectrum of PS-CH_2Cl.

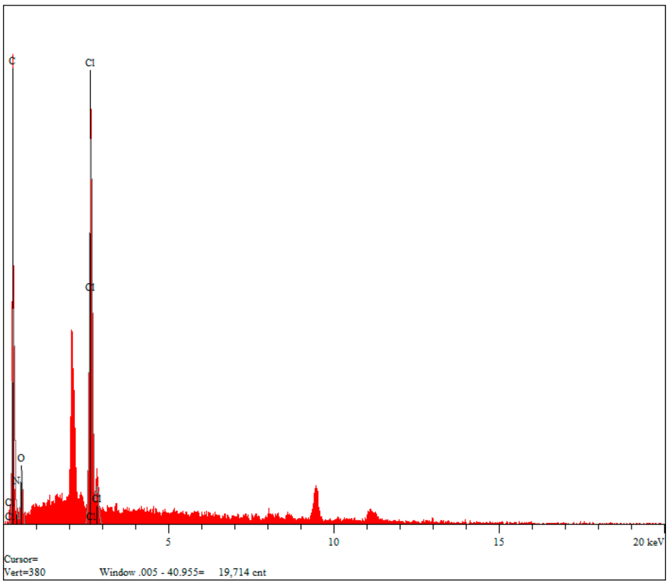

Figure S2. Energy dispersive X-ray energy spectrum of the immobilized-HBA.

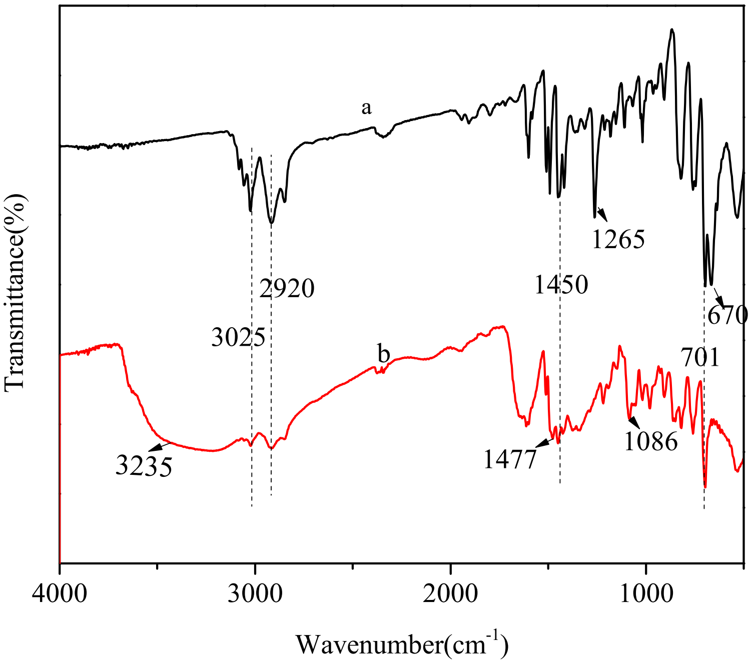

Figure S3. FT-IR spectra of PS-CH_2Cl (a) and the immobilized-HBA (b).

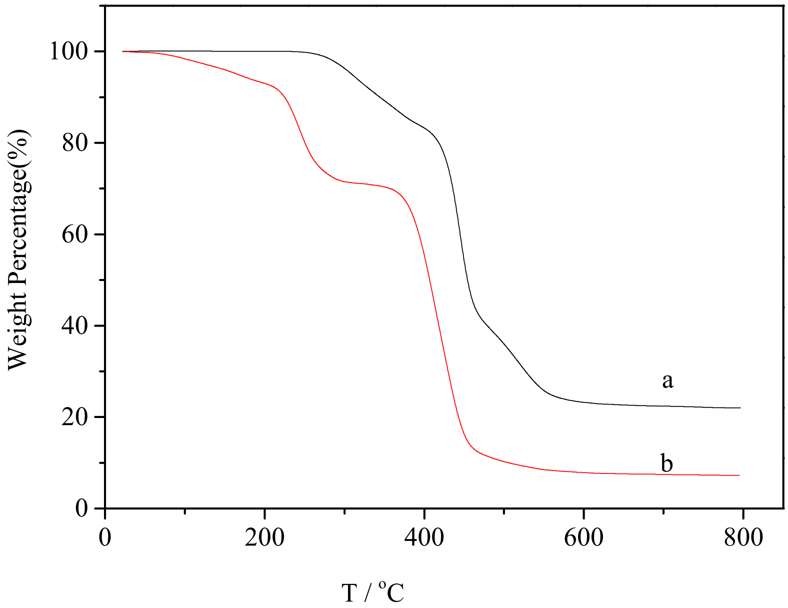

Figure S4. Thermal gravimetric analysis curves of PS-CH_2Cl (a) and the immobilized-HBA (b).

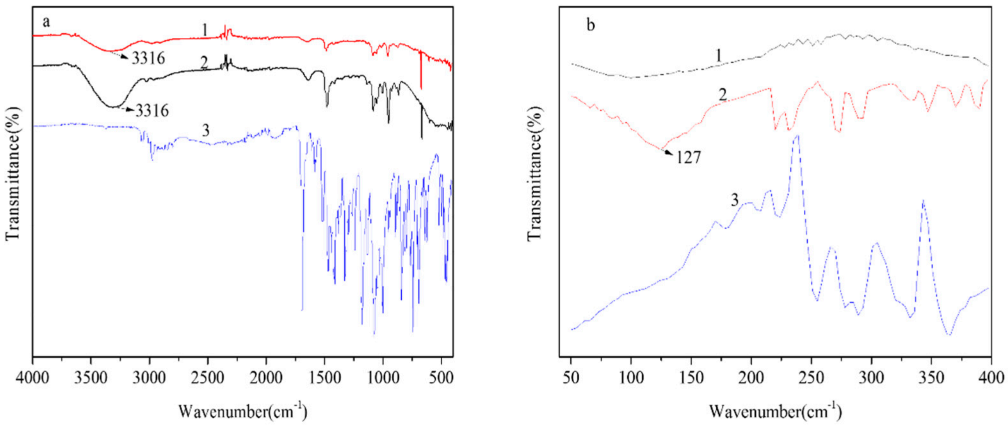

Figure S5. Mid-IR spectra (a) and Far-IR spectra (b) of choline chloride (1), choline chloride-arbidol (2), and arbidol (3).

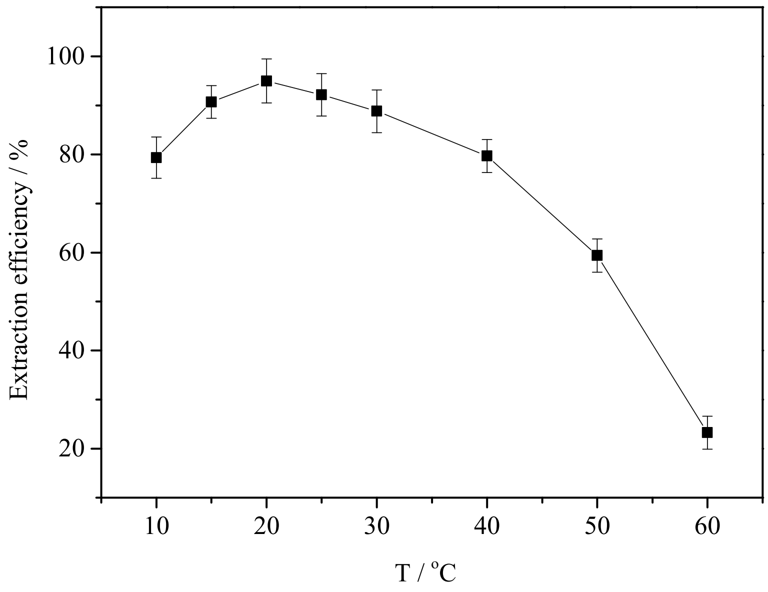

Figure S6. Influence of temperature on the extraction efficiency of arbidol. Conditions: $C_{arbidol}$ = 25 mg/L, t = 30 min.

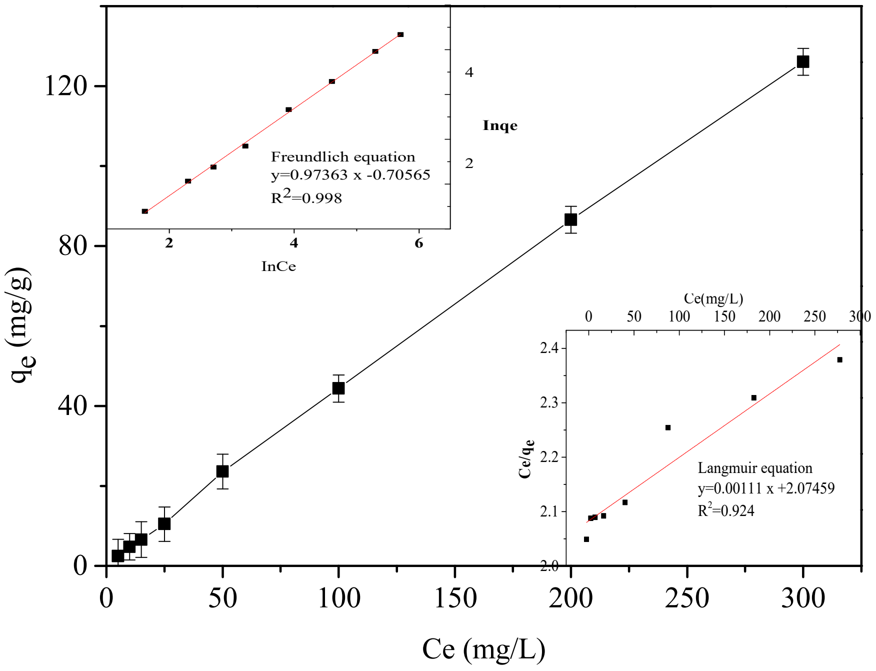

Figure S7. Adsorption isotherms of arbidol onto the immobilized-HBA fitted by Freundlich model and the Langmuir model / Conditions: t = 30 min, T = 20 °C, $m_{immobilized\text{-}HBA}$=10 mg, $V_{arbidol}$=5ml, pH=5.5.

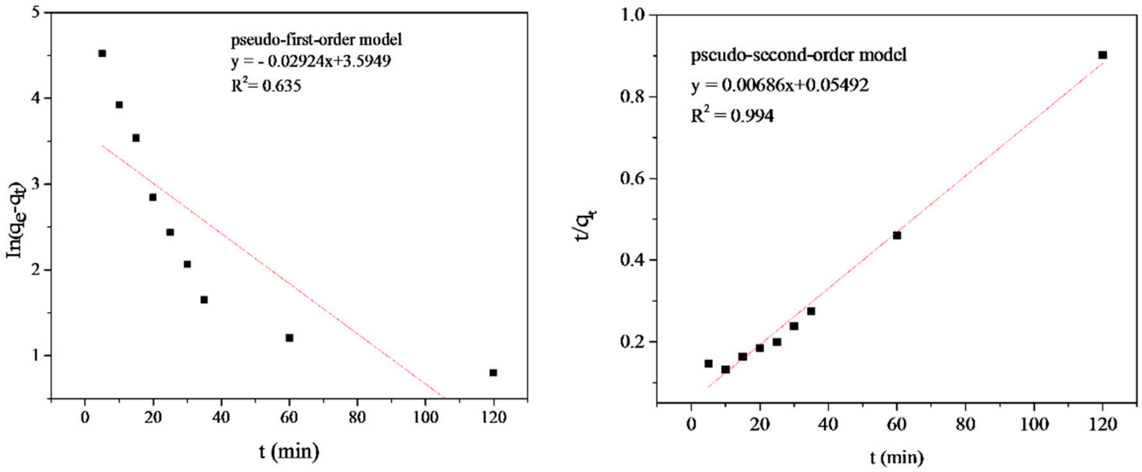

Figure S8. The pseudo-first-order model and pseudo-second-order model for the adsorption of arbidol by the immobilized-HBA / Conditions: C $_{arbidol}$ = 300 mg/L, T = 20 °C, pH=5.5.

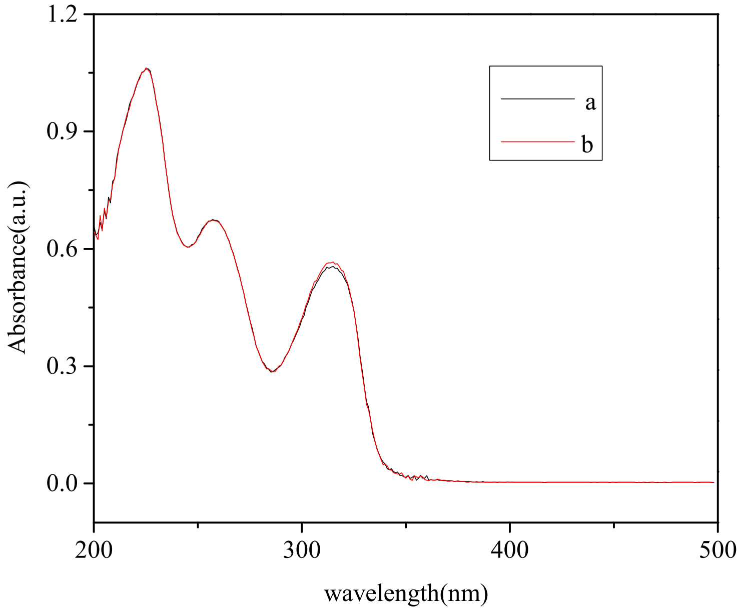

Figure S9. UV-visible absorption spectra of arbidol (a) and the recovered arbidol (b).

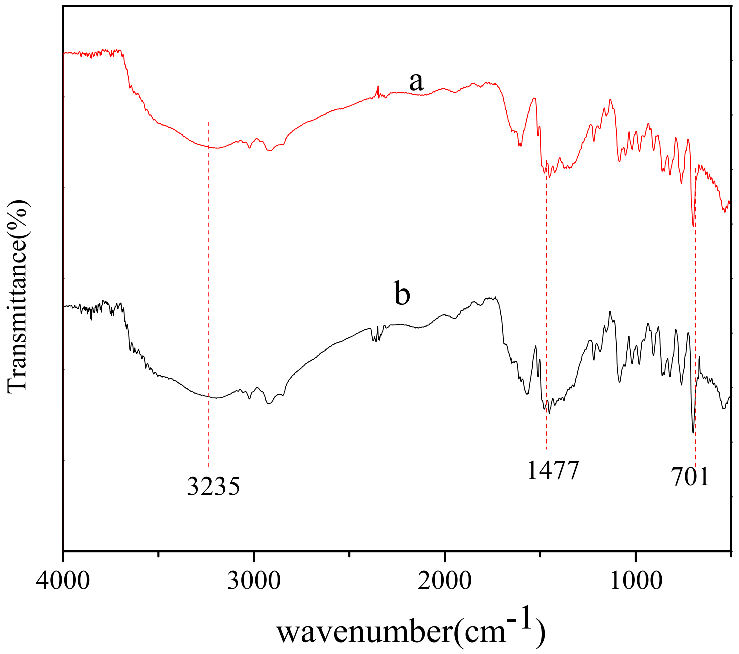

Figure S10. FT-IR spectra of the fresh (a) and the regenerated (b) immobilized-HBA.

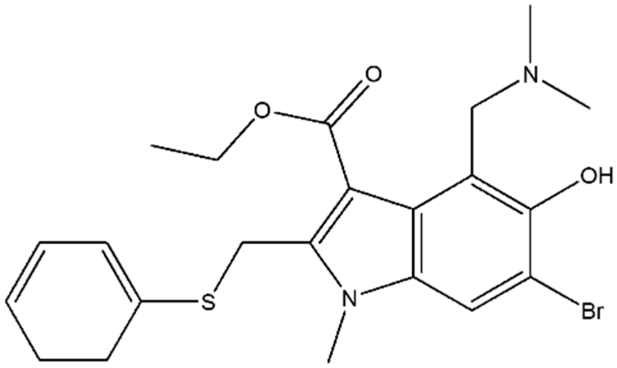

Figure S11. The chemical structure of arbidol.
